# Foreign Body Aspiration Getting Weird: Crack Pipe Aspiration

**DOI:** 10.7759/cureus.13267

**Published:** 2021-02-10

**Authors:** Chinthaka P Bulathsinghala, Palla Rivi De Silva, Rahul Dadhwal, Pahnwat T Taweesedt, Salim Surani

**Affiliations:** 1 Internal Medicine, University of Incarnate Word School of Osteopathic Medicine, San Antonio, USA; 2 Pulmonary Critical Care, University of Incarnate Word School of Osteopathic Medicine, San Antonio, USA; 3 Pulmonary Medicine, Pulmonary Associates, Corpus Christi, USA; 4 Internal Medicine, Pulmonary Associates, Corpus Christi, USA

**Keywords:** cocaine abuse, foreign body inhalation, aspiration, cough, cocaine pipe

## Abstract

Inhalation of cocaine derivatives is associated with a number of pulmonary and systemic complications. We report a case of less recognized complication, the aspiration of a metallic object used as a screen for crack cocaine abuse. A 42-year-old female presented with a two-day history of gradually worsening cough and a history of “food aspiration.” Her lung examination revealed wheezing and fine crackles with diminished air entry at the left base. A chest X-ray revealed an 8 mm radiopaque foreign body overlying the region of the left lower lobe bronchus, with a confirmatory computed tomography scan of the chest. An urgent bronchoscopy revealed a metallic foreign body impacted into the left lower lobe bronchus proper, soon after the takeoff of the superior segment, which was removed with forceps. The patient signed out against medical advice soon after. Though relatively uncommon, this case highlights a possible complication associated with crack cocaine abuse that may require emergent intervention.

## Introduction

According to the United Nations Office on Drugs and Crime, 5% of the world population aged 15 to 64 used some sort of illicit drug in 2013 [1]. As prescription pain reliever use has increased, there has been a commensurate decline in cocaine use [2]. In particular, crack cocaine is long past its boom days during the “crack epidemic,” but it is nonetheless the drug of choice for many consumers and purveyors of the illicit drug. In a 2014 report by the Substance Abuse and Mental Health Services Administration, 1.5 million people were current users of cocaine, with crack representing 354,000 of those individuals [2]. While this number has fallen over the last decade, it has been stable since 2009 [2]. Furthermore, the Center for Disease Control and Prevention (CDC) has demonstrated an association between cocaine use and heroin use, which itself has increased by more than 150% over the past 12 years [3]. Despite its decline in use, crack cocaine continues to be an important drug of abuse.

The health risks associated with crack use are not limited to the effects of the drug itself. While respiratory failure, arrhythmia, and cerebrovascular ischemia are well known to clinicians, there are consequences to its method of administration as well [4,5]. Crack pipes are often a combination of a glass stem and some sort of metallic mesh, frequently steel wool, to place the crack rock against as it is melted. These pipes are dangerous, and the British Columbia CDC has even made safer brass screens available through their Harm Reduction Program [6]. Injuries including burning of the fingers, lips, and mouth with the pipe have been reported [7]. There are case reports of aspiration of the crack pipe itself, as well as ingestion/aspiration of the steel wool material [5,6]. Here, we report a case of crack cocaine pipe aspiration in a young, healthy female.

## Case presentation

**Figure 1:**
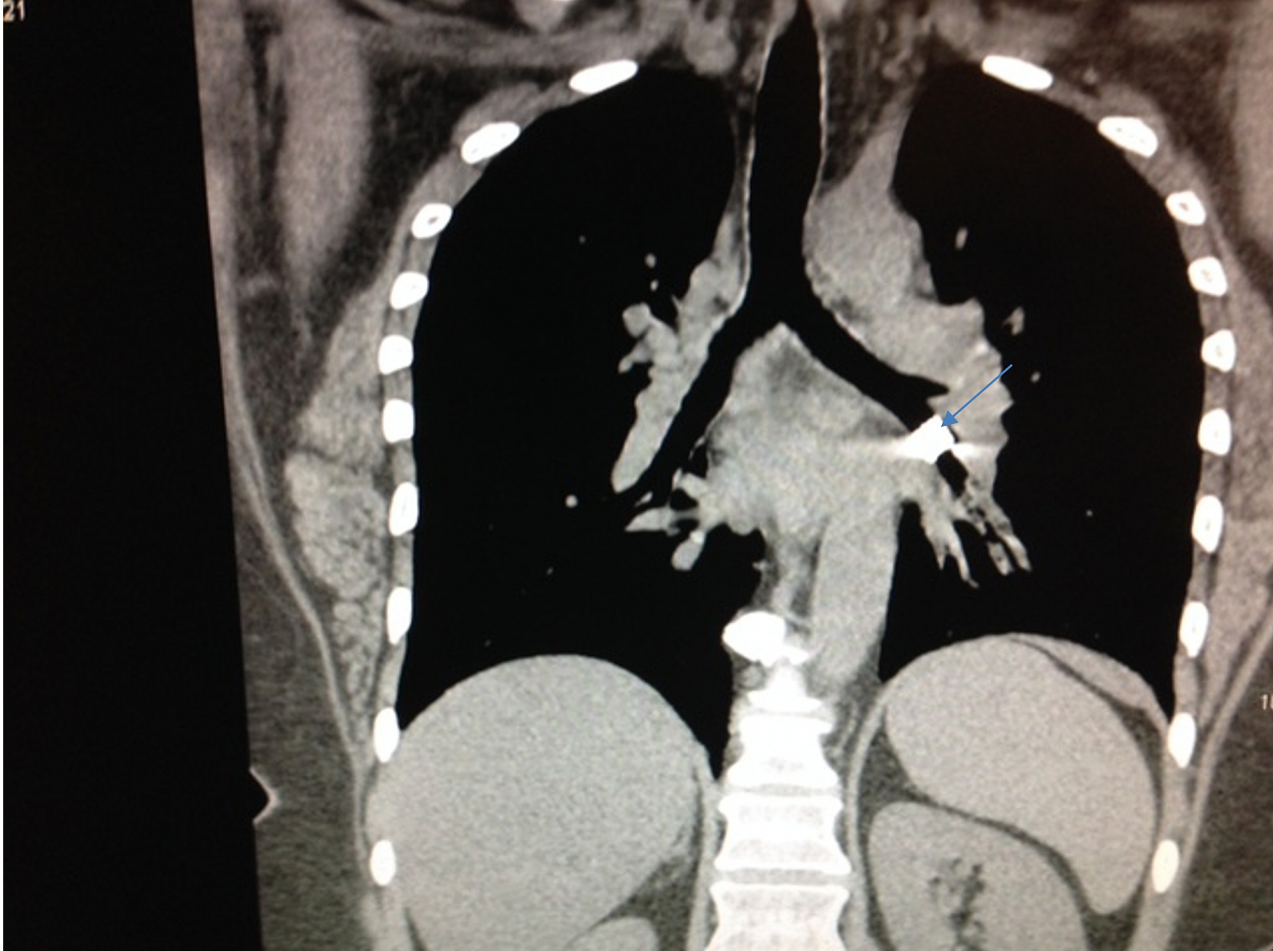
CT scan of the chest showing the metallic pipe in the left lung just after the takeoff of the upper lobe as marked by the arrow. CT, computed tomography

A 42-year-old female with a past medical history significant for asthma presented to the Emergency Department (ED) with a two-day history of cough. The cough was non-productive but gradually worsening, and she was concerned about an exacerbation of her asthma. The only other possible inciting factor she could recollect was the aspiration of food when consuming a taco about one week earlier, after which she had some neck and throat pain. Her examination revealed wheezing, fine crackles, and diminished air entry into the left base. A chest X-ray was performed in the ED, which revealed an 8 mm square-shaped radiopaque foreign body overlying the region of the left lower lobe bronchus. This was followed by a computed tomography (CT) scan of the chest, which confirmed the X-ray findings (Figure 1).

**Figure 2:**
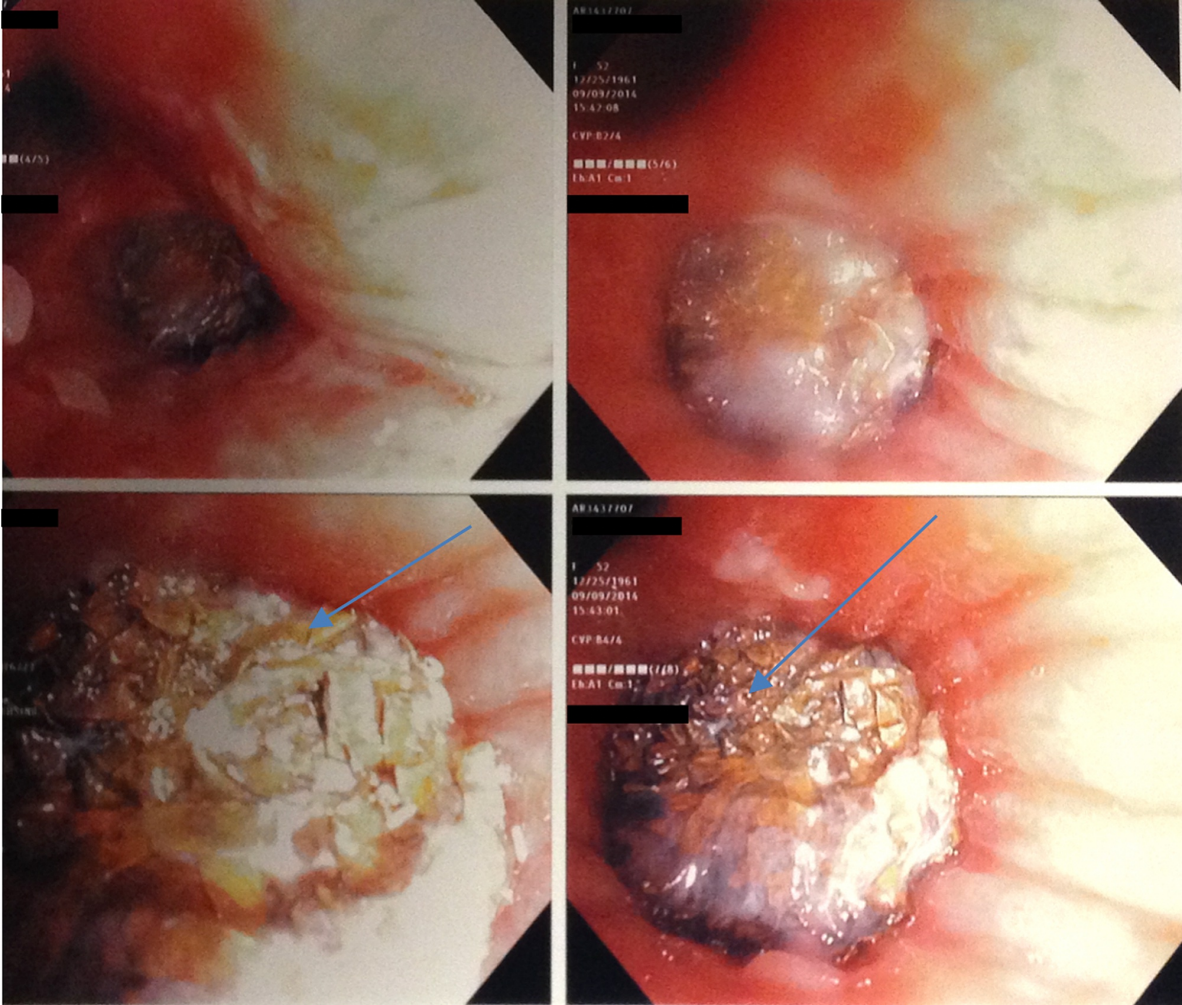
Bronchoscopic images showing the cocaine pipe in the bronchus with endobronchial forcep trying to retract it as shown by the arrow.

Additional workup was performed with complete blood count, coagulation studies, chemistries, and liver function tests, revealing only a slightly elevated white blood cell count of 12,100 per cubic millimeter. She was placed on bronchodilators and started empirically on ampicillin-sulbactam. An urgent bronchoscopy was performed, revealing the rather conspicuous metallic foreign body impacted into the lower lobe bronchus proper soon after the takeoff of the superior segment (Figure 2).

**Figure 3:**
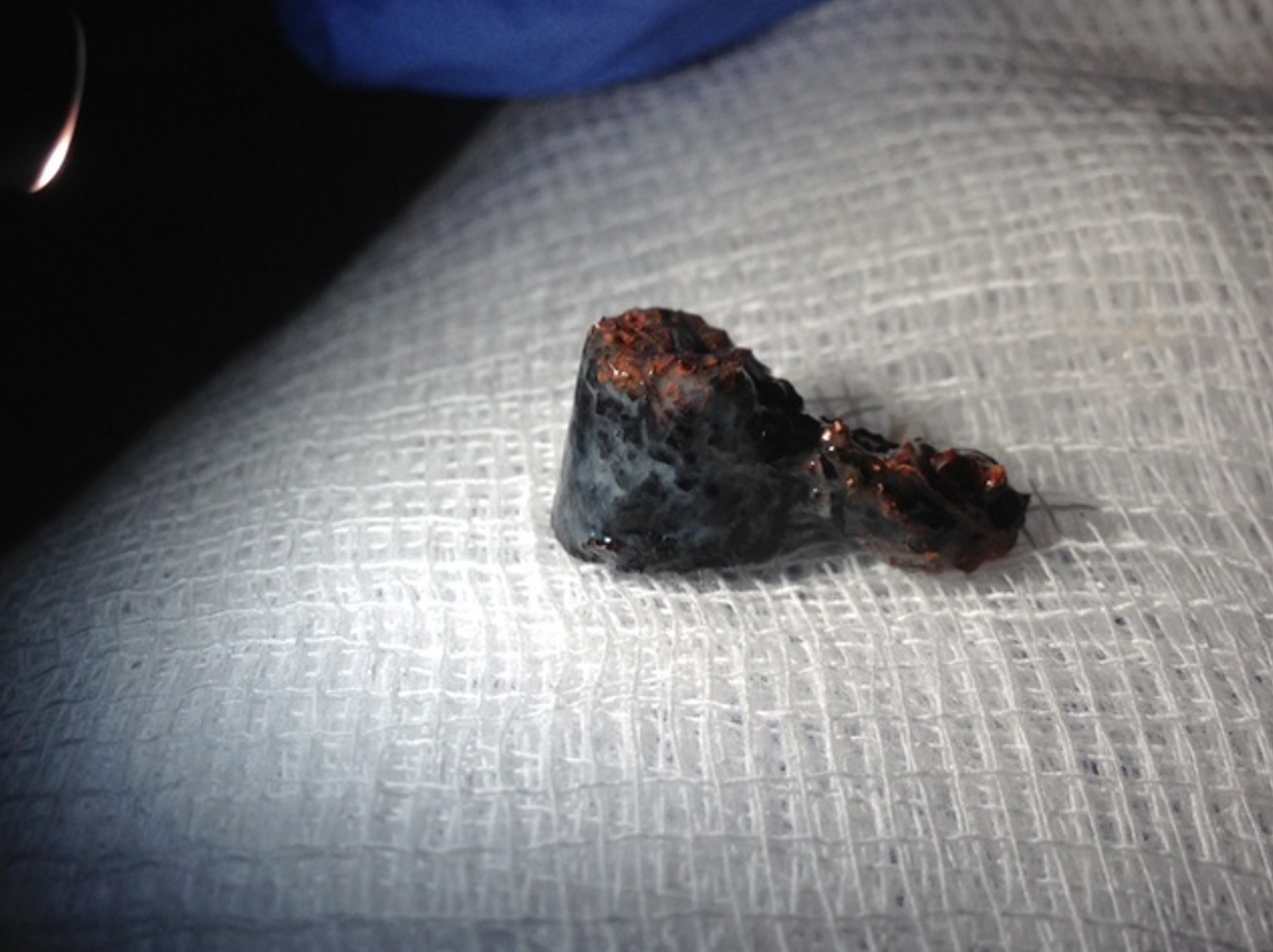
Retracted cocaine pipe form the airway.

Using only the forceps, the foreign body was grasped and withdrawn along with the bronchoscope (Figure 3).

The bronchoscope was reintroduced and inflamed bronchial mucosa was identified at the site of impaction. Unfortunately, the patient signed out against medical advice soon thereafter and did not return or follow-up.

## Discussion

Among the myriad complications associated with crack cocaine abuse, “crack lung” is perhaps the best known to clinicians and the non-medical community. The hallmark symptoms of this disease process are fever, hemoptysis, melanoptysis, hypoxemia, respiratory failure, pulmonary infiltrates, and edema [8]. By comparison, there is a relative lack of literature regarding foreign body aspiration, but it appears to be a very real threat to individuals using crack cocaine or freebase cocaine. There can also be an overlap of symptoms, including cough, hypoxemia, and respiratory failure [9]. However, some individuals may present with more chronic symptoms, including prolonged cough, fever, chills, and night sweats [10,11].

Any number of different foreign and native bodies can be aspirated from metallic objects to one’s own nasal septum [11]. Aspiration of a foreign body can lead to mechanical injury of soft tissues, burns, and even systemic sequelae [5,10]. Thus, it is important to identify what the culprit is via history and/or direct observation. However, the deleterious effects may not be limited to mechanical or thermal trauma. Depending on what the object was used for, it may be harboring any number of pathogens. These foreign bodies may also acquire pathogens as they travel through the oropharynx, as was likely the case when an aspirated hypodermic needle hub culture grew *Streptococcus viridans* and *Lactobacilli* [11]. Radiographic imaging can be useful, but identification of the offending object may not always be possible with a roentgenogram or even CT. A longstanding object might be seen as a nodular opacity, whereas recently aspirated objects may be an infiltrate [9,10]. Metallic objects should be more readily identifiable, as our case demonstrates [5].

A group colloquially referred to as “body stuffers” may present with life-threatening aspiration and ingestion. These patients ingest drugs surrounded in various forms of wrapping for the purpose of smuggling drugs, as illustrated by the McCarron and Wood classification system [12]. The task of hiding drugs within the body is sometimes performed hastily as well to avoid apprehension of contraband by authorities [13]. In either scenario, the result can be the aspiration of rubber or plastic packages with drugs inside [9,13]. Cases of cardiopulmonary arrest have even been reported due to the foreign body itself as a result of airway obstruction or drug-related toxicity as owing to packaging rupture [13,14].

## Conclusions

While aspiration of foreign bodies may not be a well-described or commonly discussed complication of crack cocaine use, it is nonetheless a concerning possibility. Our case on its most basic level plays out daily in emergency rooms nationwide with an ill patient that is uncooperative and ultimately in need of early intervention. Given the litany of different objects retrieved via bronchoscopy from patients either using or transporting illicit drugs and the lack of diagnostic consistency of non-invasive procedures, early bronchoscopic evaluation is likely warranted in symptomatic patients who report recent inhaled drug abuse. Prompt recognition and removal of the foreign body may help avoid additional damage as well as eliminating a potential nidus of infection. In those who are unwilling to discontinue cocaine use, it is reasonable to provide education regarding this complication and alternatives when available. This case report highlights the importance of proper evaluation, timely treatment, and a healthy respect for human ingenuity.

## References

[1] (2021). World Drug Report 2015. https://www.unodc.org/documents/wdr2015/World_Drug_Report_2015.pdf.

[2] (2021). Behavioral health trends in the United States: results from the 2014 National Survey on Drug Use and Health. https://www.samhsa.gov/data/sites/default/files/NSDUH-FRR1-2014/NSDUH-FRR1-2014.htm.

[3] Jones CM, Logan J, Gladden RM, Bohm MK (2015). Vital signs: demographic and substance use trends among heroin users. The United States, 2002-2013. MMWR Morb Mortal Wkly Rep.

[4] Stein M (2021). An unusual complication of crack abuse. American Journal of Medicine Blog.

[5] Moettus A, Tandberg D (1998). Brillo® pad crack screen aspiration and ingestion. J Emerg Med.

[6] Crack Pipe Screens [Internet (2021). Crack pipe screens. http://towardtheheart.com/product/crack-pipe-screens.

[7] Filho FB, da Silva YB, Martins LG, Sasso LS, Morgado de Abreu MAM (2013). Fingertip and nasal tip thermal burn in crack cocaine user. An Bras Dermatol.

[8] Mégarbane B, Chevillard L (2013). The large spectrum of pulmonary complications following illicit drug use: features and mechanisms. Chem Biol Interact.

[9] Cobaugh D, Schneider S, Benitez J, Donahoe M (1997). Cocaine balloon aspiration: successful removal with bronchoscopy. Am J Emerg Med.

[10] Libby DM, Klein L, Altorki NK (19921). Aspiration of the nasal septum: a new complication of cocaine abuse. Ann Intern Med.

[11] Lacagnina S, Comero E, Jacobson MJ, Gold AR (1990). Hypodermic needle aspiration in a freebase cocaine abuser. Chest.

[12] McCarron MM, Wood JD (1983). The cocaine 'body packer' syndrome. Diagnosis and treatment. JAMA.

[13] Narula T, Jaber W, Machuzak MS, Gildea TR (2012). Acute central airway obstruction: an occupational hazard: aspiration of crack cocaine. J Bronchology Interv Pulmonol.

[14] Pramanik P, Vidua RK (2016). Sudden cardiac death of a body packer due to cocaine cardiotoxicity. Clin Med Insights Pathol.

